# Delayed sympathetic dependence in the spared nerve injury (SNI) model of neuropathic pain

**DOI:** 10.1186/1744-8069-3-21

**Published:** 2007-07-31

**Authors:** Marie Pertin, Andrew J Allchorne, Ahmed T Beggah, Clifford J Woolf, Isabelle Decosterd

**Affiliations:** 1 Anesthesiology Pain Research Unit, Department of Anesthesiology, University Hospital Center and University of Lausanne, BH 10-CHUV, CH-1011 Lausanne, Switzerland; 2 Department of Cell Biology and Morphology, Faculty of Biology and Medicine, University of Lausanne, Bugnon 9, CH-1005 Lausanne, Switzerland; 3 Neural Plasticity Research Group, Department of Anesthesia and Critical Care, Massachusetts General Hospital and Harvard Medical School, Charlestown MA 02921, USA; 4 Centre for Neuroscience Research, Division of Veterinary Biomedical Sciences Royal (Dick) School of Veterinary Studies University of Edinburgh Summerhall, Edinburgh EH9 1QH, UK

## Abstract

**Background:**

Clinical and experimental studies of neuropathic pain support the hypothesis that a functional coupling between postganglionic sympathetic efferent and sensory afferent fibers contributes to the pain. We investigated whether neuropathic pain-related behavior in the spared nerve injury (SNI) rat model is dependent on the sympathetic nervous system.

**Results:**

Permanent chemical sympathectomy was achieved by daily injection of guanethidine (50 mg/kg s.c.) from age P8 to P21. SNI was performed at adulthood followed by 11 weeks of mechanical and thermal hypersensitivity testing. A significant but limited effect of the sympathectomy on SNI-induced pain sensitivity was observed. The effect was delayed and restricted to cold allodynia-like behavior: SNI-related cold scores were lower in the sympathectomized group compared to the control group at 8 and 11 weeks after the nerve injury but not before. Mechanical hypersensitivity tests (pinprick and von Frey hair threshold tests) showed no difference between groups during the study period. Concomitantly, pericellular tyrosine-hydroxylase immunoreactive basket structures were observed around dorsal root ganglia (DRG) neurons 8 weeks after SNI, but were absent at earlier time points after SNI and in sham operated controls.

**Conclusion:**

These results suggest that the early establishment of neuropathic pain-related behavior after distal nerve injury such as in the SNI model is mechanistically independent of the sympathetic system, whereas the system contributes to the maintenance, albeit after a delay of many weeks, of response to cold-related stimuli.

## Background

Clinicians classify neuropathic pain syndromes into either sympathetically maintained pain (SMP) or sympathetically independent pain (SIP) groups. In a subpopulation of patients (SMP group), temporary or permanent interruption of the sympathetic nervous system is associated with pain relief whereas in SIP patients, this treatment is quite ineffective [1-3]. Evidence from diverse animal models of neuropathic pain suggests the existence of a possible coupling between sensory afferent neurons and sympathetic fibers with the release of noradrenaline by sympathetic fibers exciting primary afferent neurons [4,5]. Pericellular terminal arborisations of sympathetic fibers around DRG neurons [6] are found in the DRG [7] but the number of these structures is very low in the intact DRG of naive animals [8]. Following peripheral nerve injury there is formation of a large number of pericellular baskets made of sympathetic fibers surrounding DRG neurons [9]. These sympathetic fibers may originate from vascular structures or from peripheral nerves [9,10]. These unusual novel contacts are considered a possible contributor to abnormal discharges following peripheral nerve damage, and may therefore be an integral factor in the development and maintenance of neuropathic pain [9,10].

The contribution of the sympathetic nervous system to pain sensitivity has been investigated in several animal models of peripheral nerve injury including spinal nerve ligation (SNL), chronic constriction injury (CCI) and partial sciatic nerve ligation (PSL). In these models, acute and reversible pharmacological sympathectomy or surgical sympathectomy revealed either substantial or minimal involvement with no clear explanation for the discrepancy [11-16].

We investigated whether persistent chemical sympathectomy (guanethidine injections) performed during the neonatal period of the rat affects mechanical or cold sensitivity in the spared nerve injury (SNI) model [17,18], a model of pain sensitivity in response to a distal partial nerve injury. In parallel, we investigated by immunohistochemistry the temporal evolution of sympathetic sprouting and formation of pericellular baskets in the DRG.

## Results

### Effect of neonatal chemical sympathectomy on neuropathic pain-related behavior after SNI

**Figure 1:**
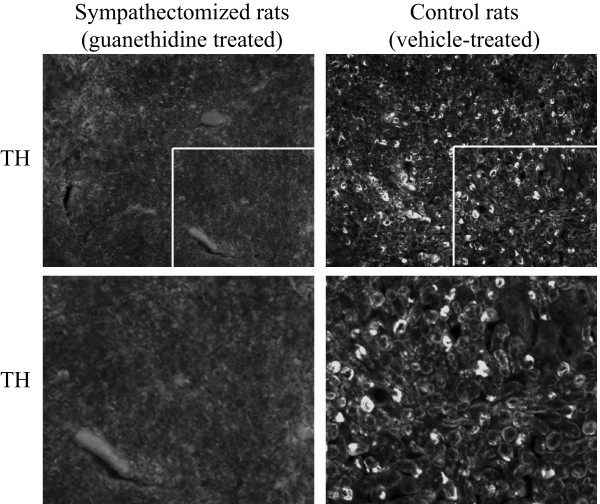
**Tyrosine Hydroxylase (TH) immunoreactivity in transverse sections of sciatic nerve (proximal to SNI injury)**. Representative microphotography of TH-IR in vehicle treated control animals (right panels), while the signal was abolished in animals injected postnatally with guanethidine (sympathectomized group, left panels).

Tyrosine hydroxylase immunoreactivity (TH-IR) was absent in 15 of the 18 rats treated with neonatal injections of guanethidine (sympathectomized groups), while an intense signal was visible in sciatic nerve sections of all vehicle treated animals (Fig 1). Data from the 3 rats in the guanethidine treated group with residual TH-IR were discarded and excluded from further analysis.

**Figure 2:**
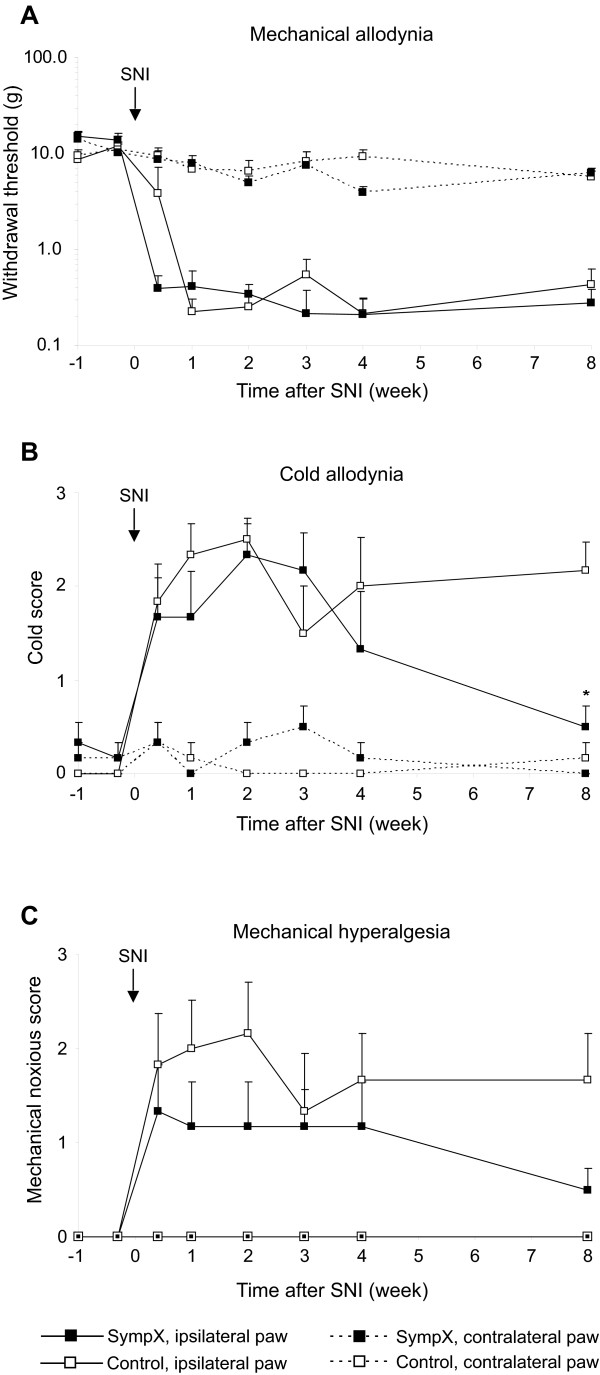
**Effect of chemical sympathectomy on the development of neuropathic pain-related behavior induced by SNI**. A. Paw withdrawal thresholds to mechanical stimuli using a calibrated series of von Frey hairs were not different between chemically sympathectomized group (sympX, n = 6) and vehicle treated group (control, n = 6) before and after SNI. B. Response to cold using an acetone drop was similar before SNI in both groups. No overall effect was demonstrated, except at the latest time point studied (* p < 0.01). C. Mechanical hyperalgesia evoked by pin prick showed no difference between groups.

In the first series of rats tested, SNI induced a significant reduction of mechanical threshold in the sural nerve skin territory in vehicle treated groups (Fig 2A), as well as an increase in cold sensitivity (Fig 2B) and mechanical hyperalgesia-like behavior (Fig 2C) compared to baseline or the contralateral hind paw (p < 0.05). In the sympathectomized animals (n = 6) the reduction in threshold and increased response to pin prick was identical to the control group (n = 6). For the response to cold a significant dissociation of the response curves between the two groups was observed at the last time point (8 weeks, Figure 2B, p = 0.01). At this time cold scores in the sympathectomized group returned to values found in the contralateral paw or baseline (p > 0.05).

**Figure 3:**
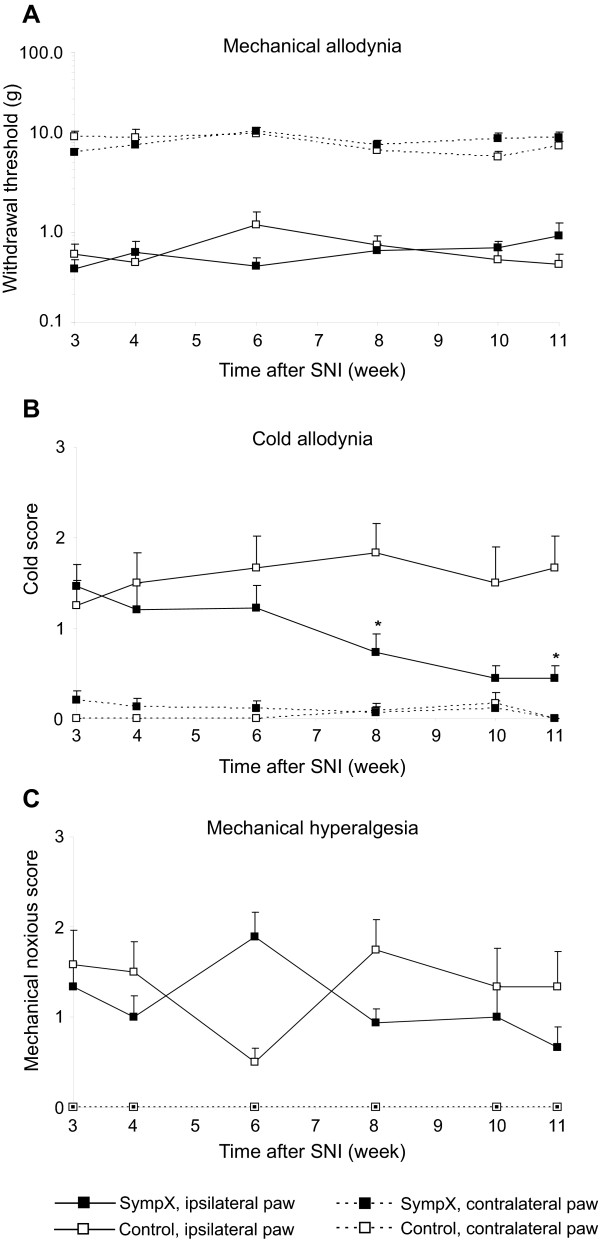
**Delayed effects on cold sensitivity of chemical sympathectomy after SNI**. A. Paw withdrawal thresholds to mechanical stimuli using a calibrated series of von Frey hairs were not different between the chemical sympathectomy group (sympX, n = 15) and vehicle treated group (control, n = 12). B. Cold response score was different between groups, lower in the sympathectomized group compared to the control group 8 weeks after SNI (* p < 0.01, n = 15 and 12, respectively) and 11 weeks after SNI (* p < 0.05, n = 9 and 6, respectively). C. Noxious mechanical response score was not different between groups. For all sensory modalities tested, responses measured in the paw ipsilateral to the SNI were significantly different from the responses of the contralateral paw (p < 0.05).

We then tested if this effect could be replicated in a longer lasting experiment in a second and independent series of rats. (Fig 3B, p < 0.05). The effect of the neonatal chemical sympathectomy was observed only on cold allodynia-like behavior, with significantly lower cold scores in the sympathectomized group 8 and 11 weeks after SNI (Fig 3B, overall effect (probability > F = 0.0008), group effect (F = 0.01), group by time effect (F = 0.0003); *post-hoc *Mann-Whitney Rank sum test: p < 0.01 at 8 weeks, < 0.05 at 11 weeks, but > 0.05 at 10 weeks.) These results suggest that the early onset of neuropathic pain-related mechanical- and cold-evoked behavior after SNI is mechanistically independent of the sympathetic system, but that the sympathetic system contributes to the maintenance of cold allodynia after a delayed period of many weeks.

For all sensory modalities tested, responses measured in the paw ipsilateral to the SNI were significantly different form the responses of the contralateral paw (p < 0.05), but no significant difference in the contralateral paw response was observed between groups (p > 0.05).

### SNI and sympathetic sprouting in the DRG

**Figure 4:**
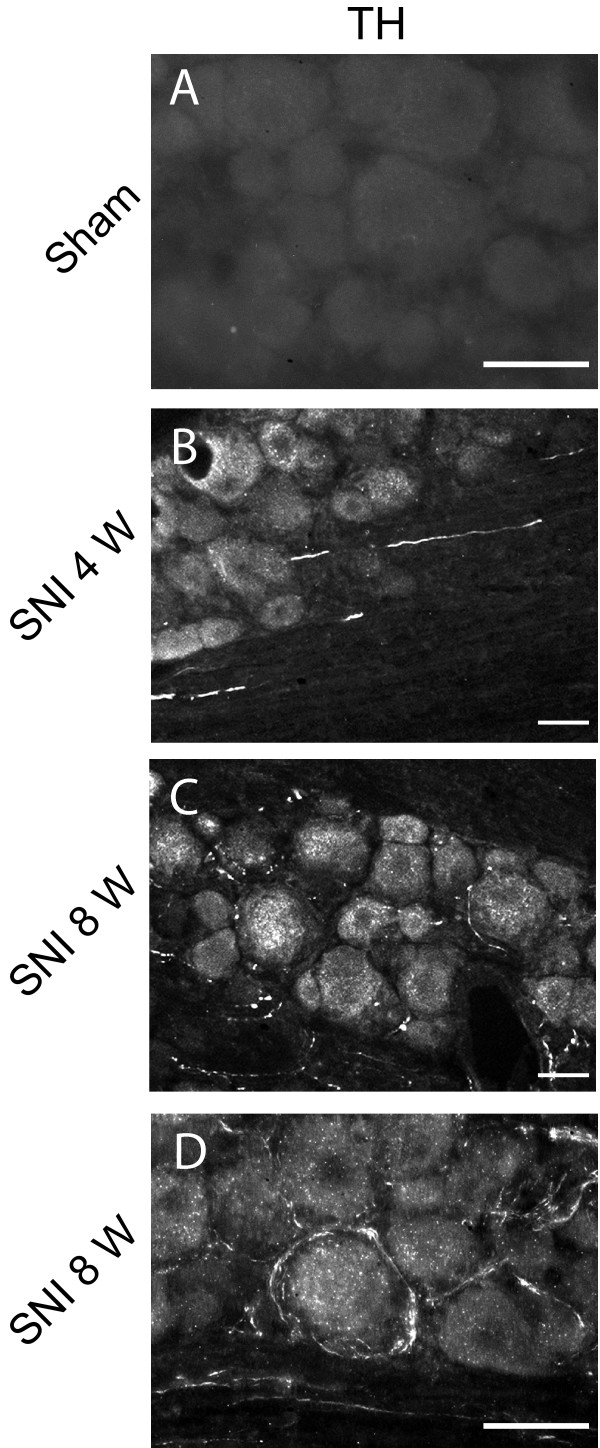
**Time course of sympathetic fiber (TH-IR positive) sprouting in L4 DRG after SNI compared to sham-treated animals**. A, No TH-IR is detectable after sham surgery. B, 4 weeks after SNI, TH-IR positive fibers co-mingle with spinal nerve fibers in the vicinity of DRG and penetrate into the DRG. C, D, 8 weeks after surgery, TH-IR positive fibers are present in the DRG and surround some DRG neurons, scale bar 25 μm.

A morphological analysis of TH-IR was performed 1, 4 and 8 weeks after SNI and sham surgery. No TH-IR was detected in the DRG or in nerve fibers close to the DRG in sham treated animals (Fig 4A) or 1 week after SNI (data not shown). Four weeks after SNI, we observed TH-IR in nerve fibers near DRG neurons, but only very few DRG neurons were surrounded by TH-IR fibers (Fig 4B). Eight weeks after SNI, TH-IR positive fibers were seen to invade the DRG and form pericellular basket-like structures around some sensory neurons (Fig 4C,D).

**Figure 5:**
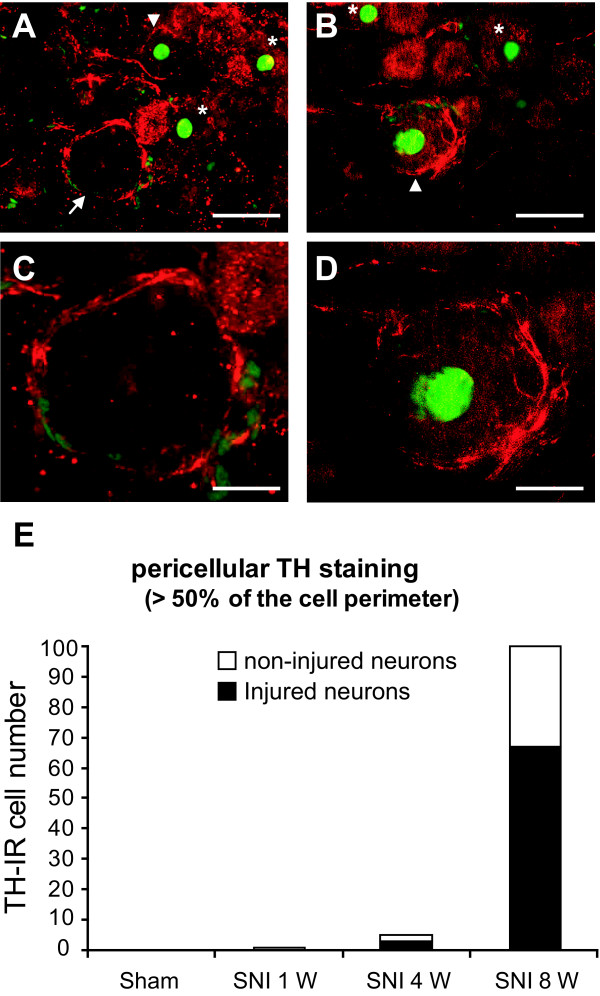
**TH-IR and pericellular baskets in injured and non-injured DRG neurons**. A to D Representative photomicrograph of double fluorescent immunohistochemistry for ATF3 (green) and TH (red) in L4 DRG 8 weeks after SNI. A and B, TH-IR fibers surround both non-injured (arrow), and injured cells (arrowhead, ATF3 positive). Not all injured neurons (asterisks) are encircled by TH-IR fibers, scale bar 25 μm. C and D, TH-IR pericellular basket structures around non-injured (C) and injured (D) DRG neurons, scale bar 10 μm. E, Quantification of TH-IR pericellular basket in injured and non-injured DRG neurons in control and SNI groups (1, 4 and 8 weeks after surgery).

We quantified these TH-IR pericellular basket structures in the DRG after SNI (n = 3 for each time point). No such structures were identified after sham surgery (0 structures out of a total cell profile of 5246 neurons) and only one TH-IR perineuronal structure was observed 1 week after SNI (corresponding to 0.02 ± 0.02%; total cell profiles 3819). Four weeks after SNI, the number of DRG neurons surrounded by TH-IR increased to 0.09 ± 0.04% of cell profiles (total profiles 4704) while at 8 weeks after SNI, the number of pericellular structures was significantly increased to 2.03 ± 0.49% (total neuron profiles 4872, p < 0.0001). The number of DRG neurons with such pericellular baskets is relatively low in the SNI model, but is very similar to that produced by other peripheral nerve injuries [19]. Using ATF3 as a marker of injured neurons, we found that 2/3 of the pericellular basket-like structures enveloped injured neurons (i.e. ATF3 positive cells, Fig 5).

## Discussion

Guanethidine sulfate produces a depletion of noradrenalin stores as well as the transmitter release and when injected subcutaneously in rats from P8 to P21, results in permanent destruction of sympathetic ganglionic neurons [20].

We find that neonatal rat sympathectomy does not detectably alter the mechanical threshold and responsiveness for almost three months after SNI, confirming observations reported for other distal nerve injury models (CCI and PSL) for various sympatholytic methodologies (pharmacological and surgical) [12,14,21,22] (Table 1). In contrast, in the SNL model, mechanical allodynia was found to be strongly dependent on the sympathetic system [22-26] (Table 1). Abolition of mechanical allodynia and heat hyperalgesia in the PSL model is dependent on the timing between the peripheral injury and the sympathetic blockade, since sympathectomy only has a strong effect 7 months after nerve injury [13]. This agrees with our finding of a reduction in cold sensitivity at 8 and 11 weeks after SNI. The delayed involvement of the sympathetic system is supported by the temporal appearance of TH-IR fibers and pericellular baskets, which only become detectable in the DRG 8 weeks after SNI. In addition, the number of DRG neurons with such pericellular baskets is relatively low in the SNI model. It has been suggested that this sympathetic sprouting inside the DRG underlies sympathetically-maintained pain [27].

**Table 1:** Effect of sympathectomy on neuropathic pain in the rat: summary of major studies in different experimental models

*Neuropathic pain model*	*sympX timing (related to nerve injury)*	*Type of sympX*	*Mechanical allodynia*	*Mechanical hyperalgesia*	*Heat hyper-sensitivity*	*Cold hyper-sensitivity*
***CCI***	5 d prior	Chemical GU		**↔^(a)^**	**↘↘↘^(a)^**	**↘↘↘^(a)^**
	Intra-operative	Surgical		**↔/↗^(b,1)^**	**↘↘^(b)^**	**↘↘↘^(b)^**
	1 w after	Surgical	**↘^(k)^**			**↘^(k)^**
	10 d after	Chemical GU		**↘^(a)^**	**↘↘^(a)^**	**↘↘↘^(a)^**
***PSL***	7 and 4 d prior	Chemical GU	**↔^(c)^**		**↘^(c)^**	
	Intra-operative	Chemical GU	**↗^(c,2)^**		**↗^(c,2)^**	
	1 w after	Surgical	**↘^(k)^**			**↘^(k)^**
	7 m after	Chemical GU	**↘↘↘^(c)^**		**↘↘↘^(c)^**	
***SNL ***L5 or ***SNL ***L5/L6	1 w prior	Surgical	**↘↘↘^(d)^, ↔^(h)^**		**↘↘↘^(d)^**	
	Intra-operative	Surgical	**↔^(g)^**			**↘^(f)^**
	Intra-operative	Chemical GU/PM	**↔^(g)^**			
	4 d after	Surgical	**↘↘↘^(f)^**		**↘↘^(d,e)^**	**↘^(k)^, ↘↘↘^(e)^**
	1 w after	Surgical	**↘↘↘^(d,k,e,i)^, ↔^(h)^, ↘↘^(j)^**		**↘↘^(d)^**	
	2 w after	Chemical GU/PM	**↘↘^(d)^**			
	3 w after	Surgical	**↘↘↘^(d)^, ↔^(h)^**			
	5 w after	Surgical	**↘↘↘^(d)^**			

^a ^[21], ^b ^[12], ^c ^[13], ^d ^[25], ^e ^[23], ^f ^[11], ^g ^[15], ^h ^[16], ^i ^[22], ^j ^[26], ^k ^[14]

^(1)^Sympathectomy induces hyperalgesia in sham animals or in contralateral hind paw, ^(2)^Possible direct effect of Guanethidine

sympX: sympathectomy, m: month, w: week, d: day

CCI: Chronic Constriction Injury, PSL: Partial Sciatic Ligation; SNL: Spinal Nerve Ligation

Sympathectomy slightly decreases (↘), decreases (↘↘), almost abolishes (↘↘↘), has no effect (↔) or aggravates (↗) pain hypersensitivity after nerve injury.

For guanethidine monosulfate (GU) and phentolamine mesylate (PM) injections:

^a ^[21]: subcutaneous injections of GU (30 mg/kg), during 4 consecutive days

^c ^[13]: one or two intraperitoneal injections of GU (30 mg/kg)

^d ^[25]: single intraperitoneal injection of GU (30 mg/kg) or PM (0.5 and 4 mg/kg)

^g ^[15]: single intravenous injection of GU (30 mg/kg) or PM (4 mg/kg)

The sympathetic sprouting in the DRG may have two distinct origins. First, SNL-type sprouting, where the nerve injury is proximal and close to both the DRG and sympathetic ganglia may induce regenerative sprouting of cut post-ganglionic sympathetic efferent neurons [10,28,29]. Second, it has been suggested that after distal lesions (SNI, SNL, CCI) collateral sprouting of undamaged sympathetic axons originating from the vasculature occurs [9,28-30]. For distal nerve injury models the sympathetic nervous system does not appear to play an essential role in the generation or establishment of neuropathic-pain symptoms; instead it only has a very restricted contribution to pain symptoms after several months, and is involved in the maintenance of only some features of pain hypersensitivity, notably cold sensitivity. Since most animal behavioral studies of neuropathic models are of short duration (2 to 3 weeks) they will miss the delayed changes that may resemble those found in patients with long established pain.

The pericellular sprouts may provide an opportunity for sensory-sympathetic coupling since it was proposed that noradrenaline released in the extracellular space in the ganglion may diffuse through the glia satellite cells and accesses α2-adrenoceptors on the neuronal soma surface [31]. Sympathetic synaptic varicosities were also observed in contact with DRG neurons surrounded by the basket structures [32]. Finally, electrophysiological experiments show that sympathetic activation modulates the DRG neuron's activity [9,33].

We found that 8 weeks after peripheral injury, pericellular baskets predominantly surround injured cells (about 70%). Several studies have attempted to characterize the signals which may account for the induction of the sympathetic sprouting (for review, see [27]). Nerve growth factor (NGF) represents a major candidate since exogenous addition of NGF but not GDNF induces basket formation [34] and antibody treatment against NGF reduces the injury-induced basket formation [29,35].

In conclusion, with the exception of cold sensitivity after two months, pain symptoms in the SNI model appear to be independent of the sympathetic system, whereas in the SNL model, pain appears to be highly dependent on sympathetic-sensory coupling. This difference between the two models highlights the fact that the extent of sensory-sympathetic coupling depends greatly on the spatial location of the nerve injury relative to the DRG, the type of injury as well as time after injury.

## Methods

### Animals, surgery and experimental groups

All experiments were approved by the animal care committee of the Massachusetts General Hospital (USA) and by the committee for animal experimentation of the canton of Vaud (Switzerland), in accordance with Swiss federal animal welfare laws and the guidelines of the International Association for the Study of Pain [36].

Male Sprague Dawley rats (Charles River Lab, Wilmington, USA and L'Abresle, France), were housed in cages under a 12 h light/dark cycle with free access to food and water. SNI surgical procedure was performed under 1.5–2.5% isoflurane (Abbott, Baar, Switzerland) general anesthesia as described previously [17]. Briefly, the common peroneal and tibial branches of the left sciatic nerve were ligated with 5.0 silk sutures (Ethicon; Johnson & Johnson, Brussels, Belgium) and transected. A 3 mm portion of the nerve was removed. Muscle and skin were sutured in two distinct layers. Sham surgery refers to the same surgical approach without injury to the nerves.

Chemical sympathectomy was produced by daily injection over two weeks of guanethidine (subcutaneously, 50 mg/kg, Sigma, St. Louis, MI, USA) in rat pups (age P8–P21). Control animals received vehicle injections. This protocol induces a permanent destruction of sympathetic neurons [20,37]. The disruption of the sympathetic system by neonatal guanethidine treatment may alter the overall physiology of the rats. However, since no differences between groups were observed for basal pain sensitivity (baseline values), this too indicates a basic integrity of the sensory system, allowing for further analyses.

At adulthood (7–8 weeks old), SNI was performed in the chemically sympathectomized and vehicle treated/control rats. Behavioral assessment was performed in a first series of sympathectomized animals and control animals (n = 6 in each group) for 8 weeks after SNI. A second series of chemically sympathectomized (n = 12) and control (n = 6) rats were tested for 11 weeks after SNI. At the end of the study, the sciatic nerve of all rats was dissected and processed for immunohistochemical analysis of tyrosine hydroxylase (TH). TH is used as a marker of sympathetic fibers since it catalyzes the conversion of tyrosine to L-DOPA, a rate limiting step in the biosynthesis of noradrenalin. A third series of rats was included in the study in order to analyze the formation of pericellular baskets in DRG after SNI. Twelve adult rats underwent surgery for either SNI (n = 3) or sham procedures and were killed 1, 4 or 8 weeks later.

### Behavioral assessment

Behavioral assessment was conducted by an observer blinded to the guanethidine treatment. Experiments were carried out by the same experimenter (AJA) in the same laboratory and in the same environment. Testing started after habituation of the rats to the experimenter and the environment. The first series of rats were tested before (2 baseline recordings) and after SNI (at 3 days and 1, 2, 3, 4 and 8 weeks after SNI). The second series of rats were tested 3, 4, 6, 8, 10 and 11 weeks after SNI. Mechanical threshold was assessed in the plantar territory of the sural nerve (the lateral plantar side of the paw) using calibrated von Frey monofilaments (Stoelting, Wood, Dale, IL, USA) in ascending order. Each filament was applied 5 times consecutively [17]. Mechanical withdrawal threshold was defined as the lowest filament (in g) that provoked a rapid withdrawal of the paw to at least in one of five stimuli. Cold sensitivity was measured by the reaction to application of a drop of acetone in the sural nerve territory of the paw. The duration of the paw withdrawal was measured (in s). The response was scored according to the following scale: 0, no visible response or startle response (< 0.5 s); 1, clear withdrawal of the paw, lasting < 5 s; 2, prolonged withdrawal of 5–10 s duration, often combined with flinching and licking of the paw; 3, prolonged repetitive withdrawal > 10 s [38,39]. Mechanical hyperalgesia was assessed by applying a brief pricking stimulus with a safety pin in the same area of the paw. The duration of the withdrawal was recorded (in s) and the response score assigned in the same manner as for the cold stimulus.

### Immunohistochemistry

Rats were terminally anesthetized with pentobarbital sodium (100 mg/kg, i.p.) and transcardially perfused with NaCl 0.9% followed by 4% paraformaldehyde in 0.1 M phosphate buffer (PB). The sciatic nerve and the DRGs were dissected, postfixed for 90 min at 4°C, and transferred to sucrose 20%, 0.1 M PB overnight. Tissues were then frozen and serially cryosectioned at 15 μm (sciatic nerve) or 12 μm (DRG). Direct fluorescent immunohistochemistry (IHC) was conducted following incubation in blocking solution (PBS, 10% normal horse serum (NHS), 0.3% Triton X-100). Sections were incubated with antibodies against TH (Santa Cruz Biotechnology, Santa Cruz, CA, USA) for 2 days (at 1:200 DRG and 1:100 nerves) in PBS, 5% NHS, 0.1% Triton X-100, followed by incubation with Cy3- or FITC-conjugated anti-goat secondary antibodies (1:300, Jackson laboratories, West Grove, PA, USA and 1:200, Vector Laboratories, Burlingame, CA, USA, respectively) in PBS containing 1% NHS, 0.1% Triton X-100. Double labeling of TH and activation of transcription factor-3 (ATF-3) was achieved by overnight incubation with anti-ATF3 antibody (1:250, Santa Cruz Biotechnology) and FITC-secondary anti-rabbit antibody (1:200, Jackson Laboratories, West Grove, PA, USA). Images were recorded by digital camera using the same conditions (e.g. exposure and gain) for the different treatment conditions (AxioCam, Zeiss, Jena, Germany). For quantification of DRG, a slide from the first 10 sections was randomly selected and then 5 sections spaced out over 60 μm were selected from the consecutive serially cut DRG sections. Immunoreactive profile for TH fibers was counted in each DRG in order to estimate the number of DRG neurons wrapped by TH-IR fibers. Every cell surrounded with TH-IR baskets that extended beyond 50% of its perimeter was measured and the presence or absence of ATF3-IR was evaluated [40]. Total number of neurons per section was also determined. DRG from 3 rats for each time point after SNI (1, 4 and 8 weeks) and after sham SNI surgery were used.

### Data analysis and statistics

Results are represented in mean ± SEM. Difference between groups for parametric values were compared using Student's *t*-test or a two-way analysis of variance (ANOVA) for repeated measures, followed by Bonferroni's post-hoc analysis when appropriate. To analyze the effect of sympathectomy on mechanical allodynia-like behavior, the transformed logarithmic values of von Frey hairs were used enabling ANOVA tests [41]. Cold and noxious mechanical scores did not follow a normal distribution and the overall effect was analyzed using Friedman repeated measures ANOVA on Ranks followed by Mann-Whitney Rank sum test for individual comparison [38]. A p value < 0.05 was considered statistically significant. Analyses were performed using JMP 5.01 software or SAS 9.1 software (SAS Institute, Cary, NC, USA).

## Competing interests

The author(s) declare that they have no competing interests.

## Authors' contributions

CJW, ID, AJA: Conceived, designed and supervised the conduct of the study.

AJA: Performed all behavioral experiments.

MP: Conducted immunohistochemistry, cell counting, Data collection and table (with ATB).

ID, MP: Analyzed the data.

ATB: Drafted the article and was responsible for editing with CJW and ID, contributing for its revision.

All the authors read and approved the final manuscript.
